# How Noise and Language Proficiency Influence Speech Recognition by Individual Non-Native Listeners

**DOI:** 10.1371/journal.pone.0113386

**Published:** 2014-11-19

**Authors:** Jin Zhang, Lingli Xie, Yongjun Li, Monita Chatterjee, Nai Ding

**Affiliations:** 1 College of Mathematics and Computer Science, Hunan Normal University, Hunan, China; 2 School of Software, Hunan University, Hunan, China; 3 Boys Town National Research Hospital, Omaha, Nebraska, United States of America; 4 Department of Psychology, New York University, New York, New York, United States of America; Utrecht University, Netherlands

## Abstract

This study investigated how speech recognition in noise is affected by language proficiency for individual non-native speakers. The recognition of English and Chinese sentences was measured as a function of the signal-to-noise ratio (SNR) in sixty native Chinese speakers who never lived in an English-speaking environment. The recognition score for speech in quiet (which varied from 15%–92%) was found to be uncorrelated with speech recognition threshold (SRT*_Q_*_/2_), i.e. the SNR at which the recognition score drops to 50% of the recognition score in quiet. This result demonstrates separable contributions of language proficiency and auditory processing to speech recognition in noise.

## Introduction

Speech recognition is robust to noise when normal hearing listeners listen to their native language, but this robustness is impaired for non-native listeners [1] and hearing impaired listeners [2]. For non-native listeners, the lack of robustness of speech recognition has been attributed to their limited ability to use phonological- and semantic-level contextual cues [1], [3]. As non-native listeners can dramatically vary in their language proficiency even when they all have normal auditory and cognitive abilities, this population can provide insights into the effects of individual listeners' language proficiency on their speech recognition in noise.

Listeners' speech recognition in noise is often quantified by a psychometric function (otherwise called a “performance-intensity function”), relating speech recognition scores to the signal-to-noise ratio (SNR). The psychometric function generally has a sigmoidal shape and can be characterized by the upper asymptote *Q*, i.e. the speech recognition score in quiet, a position parameter, i.e. the speech recognition threshold (SRT), and a slope parameter *β*. The SRT is either defined as the SNR at which the recognition score drops to 50% of *Q*, referred to as SRT*_Q_*_/2_, or the SNR at which the recognition score drops to 50% correct, referred to as SRT_50%_. Compared with native listeners, non-native listeners who have a speech recognition score near ceiling in quiet, i.e. *Q* is approximately 100%, have increased susceptibility to noise, shown by an elevated SRT [4], [5]. Thus, when comparing native and non-native speakers, language proficiency seems to affect the SRT. Within the population of low-proficiency non-native listeners whose speech recognition scores fall far below 100% even in quiet, however, it remains unclear how language proficiency influences the robustness of speech recognition in noise, e.g., as measured by the SRT.

Here, we investigate how the psychometric function relating speech recognition to noise level varies within the population of non-native listeners with low language proficiency, focusing on young normal hearing Chinese listeners who have never attended school abroad. These listeners vary significantly in their ability to recognize English, but otherwise have normal auditory and cognitive abilities. An analysis based on the individual variability within this population is used to investigate how language proficiency, as reflected by the speech recognition score in quiet, influences speech recognition in noise.

## Methods

### Listeners

Sixty listeners (19–28 years old; 32 females, 28 males) who reported normal hearing participated in this study. All the listeners were native speakers of mandarin Chinese, and were undergraduate or graduate students at Hunan Normal University, China. The experimental procedures were approved by the Degree Committee of the College of Mathematics and Computer Science, Hunan Normal University, which has the function of reviewing research involving human subjects. All listeners orally consented to participate in the study (not recorded). The data were acquired anonymously and no demographic information, except for age and gender, was acquired. No written consent was acquired. None of the listeners had majored in English or received education in English-speaking countries before participating in this study.

### Stimuli and Procedures

The recognition of English sentences was measured using the Hearing in Noise Test (HINT) sentences [6]. The sentences were presented either in quiet or mixed with spectrally matched stationary noise at −6, −2, or 2 dB signal-to-noise ratio (SNR), measured by the root mean square (RMS) value. The spectrally matched noise was generated using a 12-order LPC model derived from the HINT sentences. In each SNR condition, fifteen sentences were used. The SNR was measured based on the RMS of speech and noise.

The recognition of Chinese sentences was measured using the Mandarin Speech Perception (MSP) sentences [7]. The sentences were presented either in quiet or mixed with spectrally matched stationary noise at −13, −10, or −4 dB SNR. (Only 50 listeners were tested for the −13 dB SNR condition). Ten sentences were used in each SNR condition, and the spectrally matched noise was derived based on the MSP sentences.

The intensity of the English and Chinese sentences was normalized to be the same RMS intensity. In all SNR conditions, the intensity of the sentences was kept the same and the intensity of the noise varied. For both the English and Chinese sentence tests, sentences at different SNRs were mixed and presented in a pseudorandom order for each listener. For each speech noise mixture, the noise started 500 ms before speech, and the onset of the noise was smoothed by a 50 ms cosine window. The sentence and the noise end simultaneously. After listening to each sentence, the subjects typed in what they have heard and then started the next sentence.

The experiment was conducted in a quiet room. Stimuli were generated digitally, played via a soundcard (Realtek ALC662 HD), and presented diotically through headphones (Sennheiser HD 202). The sound volume was set at a comfortable level by the experimenter and remained the same for all listeners.

### Data Analysis

The speech recognition rate is calculated as the percent of words recognized correctly. For English recognition, a word with a morphological error, e.g. a tense error or a singular/plural error, is counted as half a wrong word. The speech recognition psychometric function obtained in each subject was fitted by a sigmoidal function of the stimulus SNR, as follows:



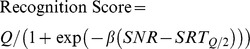



In the fitting procedure, the quiet condition is also used and the SNR is chosen to be 100 dB.

The three free parameters, i.e. *Q*, *β*, and SRT, were fitted using a maximum likelihood criterion using the Palamedes toolbox. In this equation, *Q* corresponds to the asymptotic value of the recognition score when the SNR is infinite (i.e., in quiet), *β* determines the slope of the function at SRT*_Q_*_/2_, and SRT*_Q_*_/2_, the value of the SNR at which the recognition score is at 50% of the maximum (i.e., Q/2), represents the position of the function along the × axis (SNR). The SRT_50%_ is estimated as the SNR at which the recognition score is 50%. For the purposes of the present study, the listeners' speech recognition score in quiet was assumed to reflect the level of language proficiency for each subject.

Throughout this article, a bootstrapping technique was used to assess the statistical significance of the Pearson correlation between two variables. The methods estimate the level of significance by randomly resampling the input data. The bootstrap algorithm is based on 1000 samples of the data from the 60 listeners. All statistical tests in this article are based on bootstrap estimates which are bias-corrected and accelerated [8].

## Results

### English Sentence Recognition

**Figure 1:**
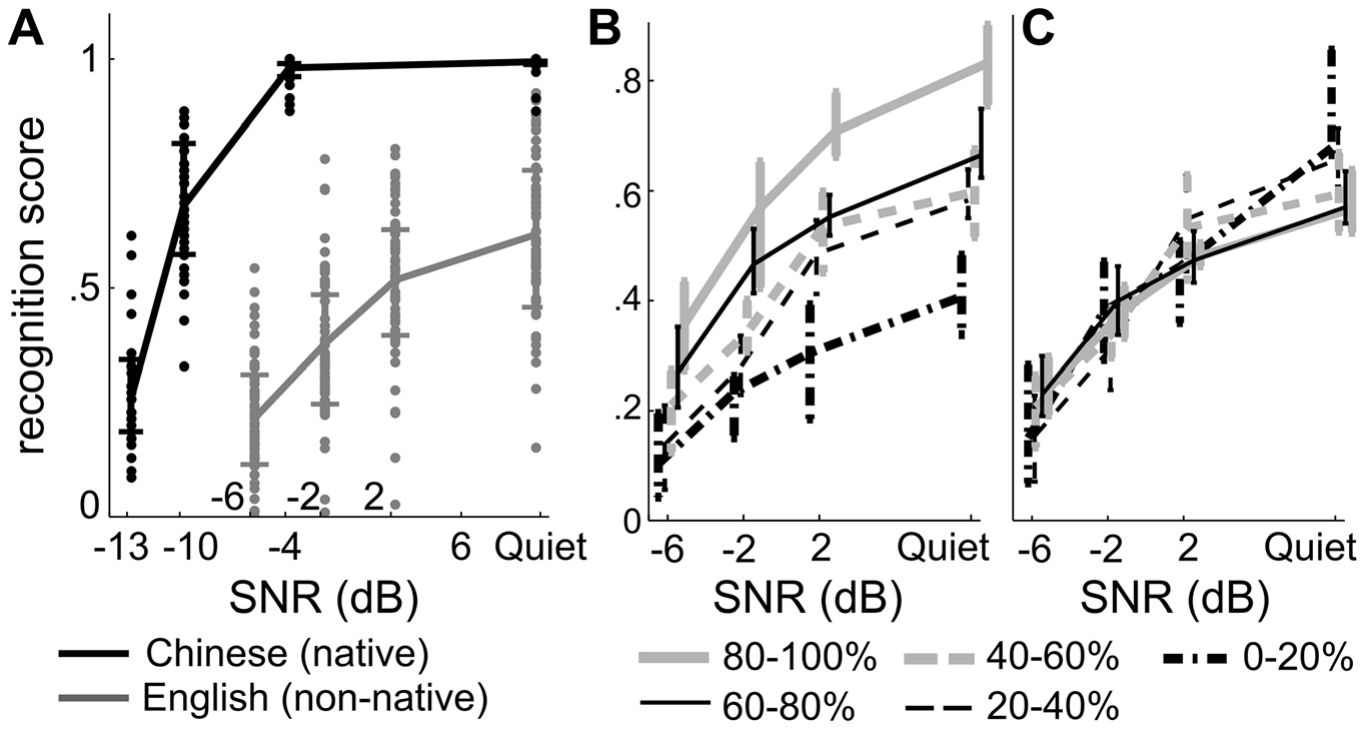
Speech recognition scores. (A) The recognition score of English and Chinese as a function of the SNR. The error bar represents one standard deviation and each dot shows data from an individual listener (B). The 60 listeners were divided into 5 groups based on their recognition score averaged over all SNR conditions, denoted as the *Q*_mean_. The psychometric functions from the 5 groups are plotted separately, and the percentile of the *Q*_mean_ for each listener group is labeled. Data from the different groups are offset slightly for clarity. (C) The psychometric functions in (B) normalized by the *Q*_mean_ averaged over each group.

The recognition score for English sentences is shown in Figure 1A for all listeners. Even in quiet, the recognition score did not reach 100% and varied widely across listeners from 15% to 92%. The recognition score was significantly correlated across stimulus conditions (Table 1). To further illustrate the individual differences between listeners with high and low English proficiency, we divided the listeners into 5 equal-size groups based on individual listeners' recognition score averaged over all SNR conditions (Figure 1B). These group-wise psychometric functions are well separated from each other, but this clear separation disappears when each function is normalized by its mean over all SNR conditions (Figure 1C). This indicates that the shape of the psychometric function is not strongly affected by the recognition score in quiet. The only noticeable difference between listener groups is that listeners with a higher averaged recognition score tend to have a psychometric function with a shallower slope (Figure 1C). To further quantify the differences in the psychometric functions observed across listeners, we fit the sigmoidal function for each listener as described under Methods.

**Table 1:** The correlation coefficient between the speech recognition score in different listening conditions.

		English (EN)			Chinese (CN)			
		−2 dB	2 dB	Quiet	−13 dB	−10 dB	−4 dB	Quiet
EN	−6 dB	**0.71**	**0.58**	**0.51**	0.01	0.10	0.09	0.12
	−2 dB		**0.68**	**0.57**	−0.12	0.07	0.08	0.10
	2 dB			**0.67**	−0.14	0.14	0.22	0.01
	Quiet				−0.04	0.01	0.18	−0.02
CN	−13 dB					0.09	−0.11	−0.11
	−10 dB						0.12	0.08
	−14 dB							0.08

Statistically significant correlations are shown in bold (*P*<0.0002, no correction applied for multiple comparison, bootstrap). Other correlations do not reach the significance level (*P*>0.5, boostrap).

The fitted *Q* was significantly correlated with the speech score in quiet (*R* = 0.97, *P*<0.001). On average, the fitted SRT*_Q_*_/2_ was −4.1 dB and *β* was 0.76. The SRT*_Q_*_/2_ was not significantly correlated with either *Q* (*R* = −0.04, *P* = 0.25) or *β* (*R* = 0.01, *P* = 0.41). However, there was a weak but statistically significant negative correlation between *β* and *Q* across listeners (*R* = −0.23, *P* = 0.027). This result confirms the observation in Figure 1C that the psychometric functions of listeners with higher speech recognition scores have a slightly shallower slope. For a subgroup of listeners (*N* = 46) whose speech score was above 50% in quiet and below 50% for the lowest SNR condition, we also estimated the SNR at which the fitted psychometric function reaches 50%, i.e. SRT_50%_. The SRT_50%_ was 0.6 dB on average and was significantly correlated with *Q* (*R* = −0.48, *P*<0.001) and *β* (*R* = 0.16, *P* = 0.005).

### Mandarin Chinese Sentence Recognition

The recognition of Chinese sentences is shown in Figure 1A. The recognition score was not significantly correlated across any two stimulus conditions (Table 1). The psychometric function for each listener was fitted by the same sigmoidal function described above. On average, the fitted SRT was −11 dB and *β* was 0.69. *Q* was saturated near 1.0 for all listeners, and SRT was not significantly correlated with *β* across listeners (*R* = −0.0076, *P* = 0.49).

## Discussion

This study investigated how the speech recognition psychometric function is affected by language proficiency within a population of non-native speakers. In particular, we focused on English sentence recognition by native speakers of Mandarin Chinese who have never lived in English-speaking environments. Results showed that language proficiency (*Q*) has a modest but statistically significant influence on the slope of the psychometric function (*β*) and has no significant effect on its position (SRT*_Q_*_/2_).

A few distinctions are seen when the same subject group listens to native and non-native languages. First, for a non-native language, the speech recognition score is correlated between SNR conditions (Table 1), consistent with findings from a previous study [9]. This indicates that the recognition score is similarly affected by a common factor in all SNR conditions. It seems reasonable to speculate that this common factor is language proficiency. For the native language, however, no such significant correlation is seen between any two conditions. Second, when the recognition score is similarly low for the Chinese and English listening tasks (the −13 dB and the −6 dB condition respectively), no strong correlation is seen between the recognition scores in the two conditions. One possible reason is that, in a low SNR condition, the recognition score for the native language depends mostly on auditory processing while the recognition score for the non-native language depends on both auditory and language processing. Another possibility is that the auditory mechanisms involved in speech processing depend on the SNR [10] even when the speech recognition scores are matched.

Although a difference in the SRT*_Q_*_/2_ was not observed in this study for the listeners differing in Q, a change in SRT_50%_ is often observed when comparing native and non-native listeners in sentence recognition tasks [4], [5]. In these previous studies, the non-native listeners had near-ceiling speech recognition scores in quiet, and SRT_50%_ roughly equaled SRT*_Q_*_/2_. As the non-native listeners' performance in quiet was near ceiling, it is possible that the effect of language proficiency appeared only in low SNR conditions. As a result, in the psychometric function, language proficiency affected the SRT_50%_ or SRT*_Q_*_/2_. In the lower language proficiency group tested in the present study, however, the speech recognition score remained below ceiling even in quiet, providing the opportunity to demonstrate that language proficiency does not interact with listeners' speech recognition in noise.

In summary, this study characterized the psychometric function of the speech recognition scores of young, native Chinese-speaking listeners recognizing a non-native language (English) in noise. Language proficiency (as reflected by the speech recognition score in quiet) showed a strong influence on the upper asymptote of the psychometric function, very weak influences on its slope, and no influences on its position. We infer that the slope and position of the psychometric function are likely to be determined by auditory functions such as the ability to separate speech from noise, whereas the upper asymptote relates to factors such as verbal and linguistic knowledge.

## Supporting Information

Materials S1 (MAT)
